# The Green Planet: development and early evaluation of a novel method for climate actions in clinical practice

**DOI:** 10.1186/s12913-026-15059-z

**Published:** 2026-07-02

**Authors:** Linda Sturesson Stabel, Desirée Kristensson, Henna Hasson, Greta Schettini, Charlotta Sävblom, Pamela Mazzocato

**Affiliations:** 1 Department of Learning, Informatics, Management and Ethics, Medical Management Centre, Karolinska Institutet, Stockholm, Sweden; 2 Centre för Epidemiology and Community Medicine, Region Stockholm, Stockholm, Sweden; 3 Department of Clinical Neuroscience, Centre for Psychiatry Research, Karolinska Institutet & Stockholm Health Care Services, Region Stockholm, Stockholm, Sweden; 4 Centre for Dependency Disorders, Stockholm Health Care Services, Region Stockholm, Stockholm, Sweden; 5 Södertälje Hospital, Södertälje, Sweden

**Keywords:** Climate change, Environment, Quality improvement, Leadership, Delivery of healthcare, Qualitative study, Health personnel, Social change, Behaviour change, Service design thinking

## Abstract

**Background:**

Climate change is becoming an increasingly serious threat to human health. At the same time, healthcare delivery is contributing substantially to greenhouse gas emissions, thus aggravating climate disruption. To integrate climate actions into clinical practice is therefore important but remains challenging. Health professionals often express environmental awareness but face barriers to engaging in pro-climate behaviours at work. There is limited evidence on how workplace interventions can be designed and implemented to support such behaviours. This study describes the development and early evaluation of Green Planet, a co-created method designed to integrate climate awareness and actions into healthcare.

**Methods:**

The Green Planet was developed through a service design process in collaboration with managers and clinical staff at a Swedish hospital, adapting an established quality improvement method, the Green Cross, to focus on climate actions. The method was tested over a six-weeks period in a medicine ward. A qualitative evaluation was conducted using eight semi-structured interviews, one focus group discussion with eleven participants, and three non-participatory observations. Data was analysed using an inductive thematic analysis to examine how the method was used in practice, and a deductive thematic analysis guided by the Capability, Opportunity, Motivation–Behaviour (COM-B) model to explore how the intervention influenced conditions for pro-climate behaviours.

**Results:**

The Green Planet was largely implemented as intended, with local adaptations to fit clinical routines. Staff reported that the method increased awareness and knowledge about climate actions, promoted reflection on everyday practices, and fostered a sense of collective responsibility. The intervention enhanced capability, opportunity, and motivation for pro-climate behaviour, particularly by making climate actions visible, concrete, and part of daily team discussions. Challenges included variable staff engagement, time constraints, and reliance on a designated test leader.

**Conclusions:**

This early evaluation suggests that a co-created, theory-informed method integrated into existing work routines can strengthen conditions for pro-climate behaviours in healthcare settings. The Green Planet shows promise as an adaptable approach to normalising climate action in everyday clinical practice. Further research is needed to assess long-term effects, scalability, and measurable environmental impact.

**Supplementary Information:**

The online version contains supplementary material available at 10.1186/s12913-026-15059-z.

## Background

The harmful effects of climate change are becoming increasingly prominent and pose a major threat to human well-being, health, and survival [1, 2]. The healthcare sector plays a crucial role in meeting population health needs but is paradoxically responsible for 4.2% of global greenhouse gas emissions [1]. Hospitals are major contributors to these emissions [2, 3] as buildings and technical equipment are energy-intensive around the clock [4], and large quantities of products and pharmaceuticals, including high-impact medical gases, are used [5].

To limit emissions, a variety of decarbonisation actions have been proposed, such as changes in clinical practices (e.g. reducing low-value care), improved waste management (e.g. minimizing single use items), energy conservation (e.g. turning off unused equipment), and reducing travel and transportation [6–9]. However, despite broad recognition of their importance, translating these actions into daily practice remains challenging. While healthcare professionals may demonstrate environmental awareness [10] and willingness to engage in climate actions [11], considerably fewer take such actions in the workplace [10, 11]. For instance, a recent study on nurses indicated that while 70% demonstrated environmental awareness, 52% rarely engaged in workplace climate actions, and 35% found them difficult to implement [10].

The persistent gap between climate awareness and action in healthcare [10, 12, 13] highlights the challenge of fostering pro-climate behaviours (PCBs) in clinical settings. Behaviour change is inherently complex to initiate and sustain [14–16], and although several behaviour change interventions have been tested to support PCB in healthcare [17, 18], PCBs remain understudied compared with those in other sectors, such as hospitality and manufacturing [19]. Evidence on how to design effective behaviour change interventions to reduce the carbon footprint of the healthcare sector remains limited [17, 18], underscoring the need to better understand how such interventions can create conditions for PCBs in clinical practice.

### Pro-climate behaviour changes: barriers and interventions

Several barriers hinder healthcare professionals from engaging in PCBs. Healthcare professionals have reported a lack of time, resources, methodological support, and knowledge needed to succeed with climate actions [12, 20–24]. Additional challenges include unclear responsibilities, limited stakeholder commitment, and conflicting priorities, e.g. the perception that climate actions may jeopardize quality of care [24–26].

Less is known on how to design interventions to overcome these barriers [17, 18]. One review on clinical and health services interventions aimed at reducing greenhouse gas (GHG) emissions, however found that interventions targeting clinician behaviour, such as education, clinical practice guidelines, and feedback, were most commonly reported, followed by delivery arrangements to improve greener clinical care, such as changes to reduce anaesthesia use and conversion from disposable to reusable equipment [18]. Another review, focusing specifically on interventions targeting behaviour change among clinicians identified only six articles published in peer-reviewed journals [17]. The most commonly used behaviour change interventions included social support, salience of consequences, restructuring of the physical environment, prompts and cues, feedback on outcomes of behaviour, and information about environmental consequences [17]. Both reviews however concluded that, despite some positive effects of behaviour change interventions targeting PCBs in healthcare, the evidence regarding their effectiveness remains uncertain [17, 18]. Moreover, they highlighted the need for theory-informed development and evaluation of behaviour change interventions, as well as a clearer reporting of intervention components [17, 18]. As most research on PCBs in healthcare has focused on anaesthesia care [17, 18], there is also a need to generate evidence from a broader range of healthcare settings, such as hospital wards.

### Theoretical perspectives to develop behaviour change interventions to support PCBs

Research in implementation science provides key principles for designing effective behaviour change interventions to foster PCBs in healthcare. Central is the understanding that behaviour change depends on three essential conditions, conceptualised as three interconnected components: individuals need to have the *capability* to perform the behaviour, the *opportunity* to engage in it, and the *motivation* to enact and sustain it [27, 28]. More specifically, *capability* encompasses both physical (e.g. skills and ability) and psychological (e.g. knowledge and mental state) components. *Opportunity* refers to the context, including the physical environment (e.g. time and financial resources) and social influences (e.g. norms, support, and peer pressure). *Motivation* includes both automatic processes (e.g. habits or emotional responses) and reflective processes (e.g. planning and decision-making) that influence behaviour [28]. Together, these interrelated conditions are conceptualized within the COM-B model of behaviour. The COM-B model is a widely used framework for identifying and understanding individual, socio-cultural, and contextual influences on behaviour [28]. It has previously been applied to identify influencing factors for environmentally sustainable behaviour in healthcare settings [29]. For example, a study of gynaecological surgery found that unsustainable practices were linked to inadequate knowledge of sustainable methods, limited leadership support, staff attitudes, and restricted access to sustainable medical devices [29]. These findings highlight the need to design interventions that support PCBs by systematically addressing the three COM-B conditions [17].

Further, organisational studies suggest that effective workplace interventions should be co-created with end users and built on existing routines and processes [30]. It has been emphasized that managers and staff should not be passive recipients of organizational interventions [30], but instead actively involved in shaping, managing, and taking ownership of them [31]. Co-creation processes take into account local contextual conditions, needs, and goals during the development of interventions [32], thereby enhancing stakeholder engagement [33, 34]. Design thinking [35] is a widely used method for co-creation in healthcare [36, 37], including in the design of interventions aimed at reducing GHG emissions in the sector [38, 39]. Design thinking is a human-centred, creative problem-solving approach that relies on iterative cycles of ideation, rapid prototyping, and testing [35] to develop innovative solutions to complex problems. By incorporating the needs of those experiencing a problem, design thinking can improve the uptake of interventions aimed at fostering PCBs.

## Rationale and aim of the study

Despite growing awareness of healthcare sector’s impact on the climate, healthcare professionals face substantial barriers to engaging in PCBs, and effective, theory-informed behaviour change interventions remain limited. Implementation and co-creation frameworks, such as COM-B and design thinking, offer promising approaches for developing interventions that are better aligned with clinical contexts. This study therefore explores how a co-created intervention can be used and how it may influence the conditions that enable PCBs. The specific research questions (RQs) are as follows:

### RQ1

How can a co-created intervention to promote PCBs be used in clinical practice and how do managers and staff experience its use?

### RQ2

How do managers and staff experience a co-created intervention to influence the conditions, i.e. capability, opportunity, and motivation, to engage in PCBs?

## Methods

### Study design

This study is part of a larger project that employed service design [35, 40] to co-create practical interventions to promote PCBs in a Swedish hospital. The study focuses on one of the developed behaviour change interventions and uses qualitative methods to explore how it was used and experienced. The SQUIRE (Standards for Quality Improvement Reporting Excellence) guidelines were used for reporting [41].

### The context

In Sweden, the national government is responsible for overall policies, while the responsibility for financing, organising and providing healthcare is delegated to local regions. Regional agreements, aligned with the national mission of achieving net-zero emissions by 2045 [42], mandating that hospitals actively work to reduce their environmental impact.

The study was conducted at a hospital with 1300 employees and 200 beds, operating as a combined emergency and community hospital offering a wide range of acute and planned services. The hospital had been certified under an environmental management system (ISO 14 001) since 2005. A sustainability strategist supported the senior management team in defining actions to achieve environmental goals. At the unit level, 50 clinical staff members held roles as environmental representatives, supporting practical actions. All employees were expected to complete a 60-minute online course on sustainability.

### Co-creation of the intervention

**Fig. 1:**
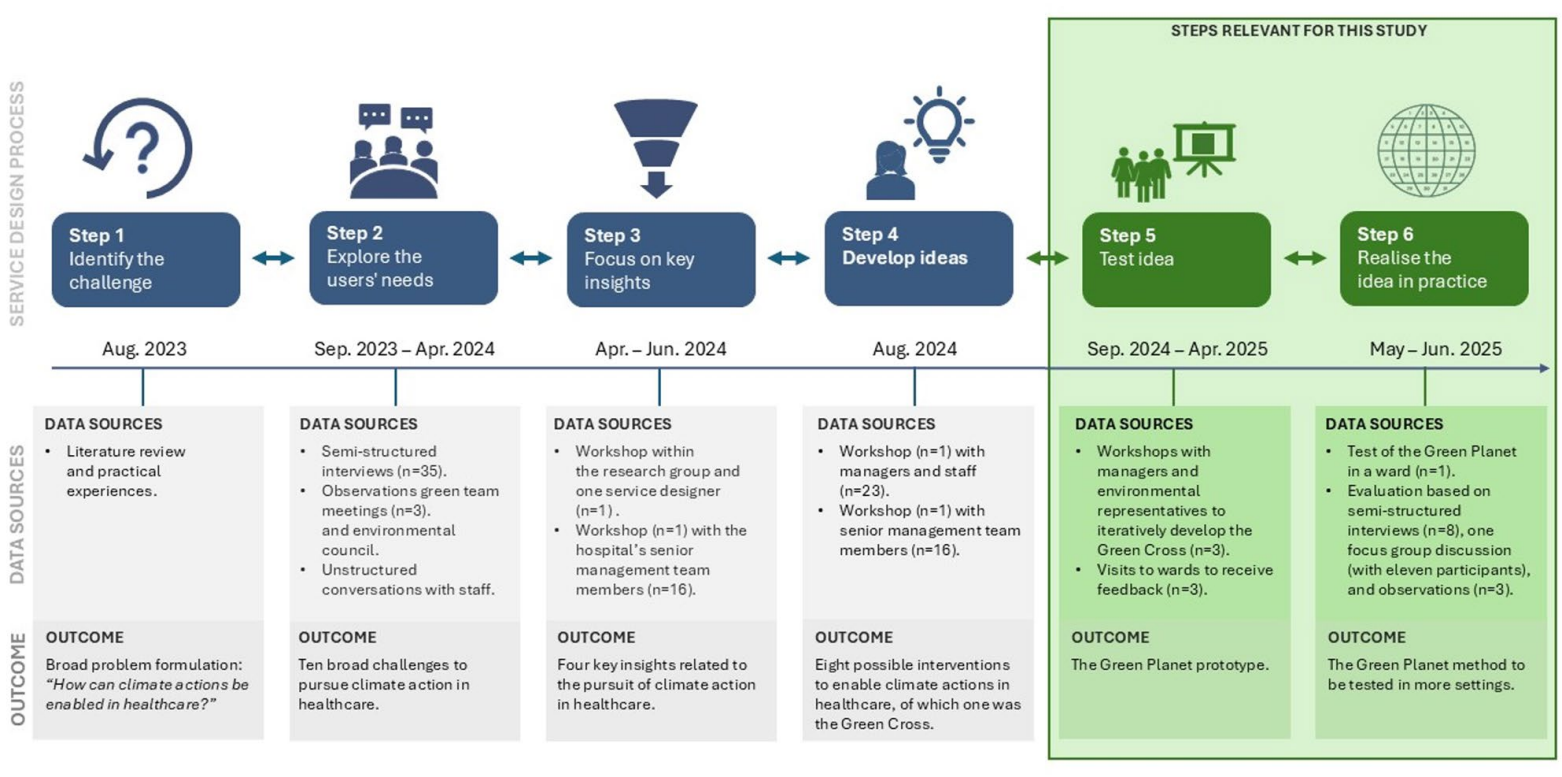
Service design process employed

The service design process was led by an experienced service designer with a nursing background, in collaboration of the first (LSS) and last (PM) authors, and with contribution from the second (DK), third (HH), fourth (GS), and fifth (CS) authors. The process, illustrated in Fig. 1, began in 2023 by defining the problem—namely, that managers and clinical staff experienced difficulties in implementing climate actions—and exploring their experiences of this challenge (steps 1–2). Several challenges were identified and subsequently narrowed to four key insights (step 3): climate issues were perceived as separate from clinical work; leaders adopted a cautious approach to climate actions; the hospital’s climate impact and actions had limited visibility; and climate efforts were easily deprioritised. In step 4, ideas to address these issues were generated during a workshop involving managers and environmental representatives. One proposed solution was to apply an existing quality improvement method, the Green Cross [43] (see below), to support climate actions. In step 5, the Green Cross was adapted and further developed into the Green Planet through a process that included workshops with managers and clinical staff serving as environmental representatives, site visits at hospital wards, and a review of relevant research literature. In step 6, the Green Planet was tested and evaluated using qualitative methods. For this study, steps 5–6 are relevant.

### The intervention: the Green Planet

The original Green Cross intervention is a simple visual method developed in manufacturing and construction industry known as the Safety Cross. The method has been adapted for use in healthcare, where it is known as the Green Cross, to enable professionals to recognize safety incidents [43]. It is a team-based approach in which incidents are discussed during daily safety briefings and weekly quality improvement meetings [44], with promising results in patient safety culture [43], work engagement, and teamwork climate [45].

**Fig. 2:**
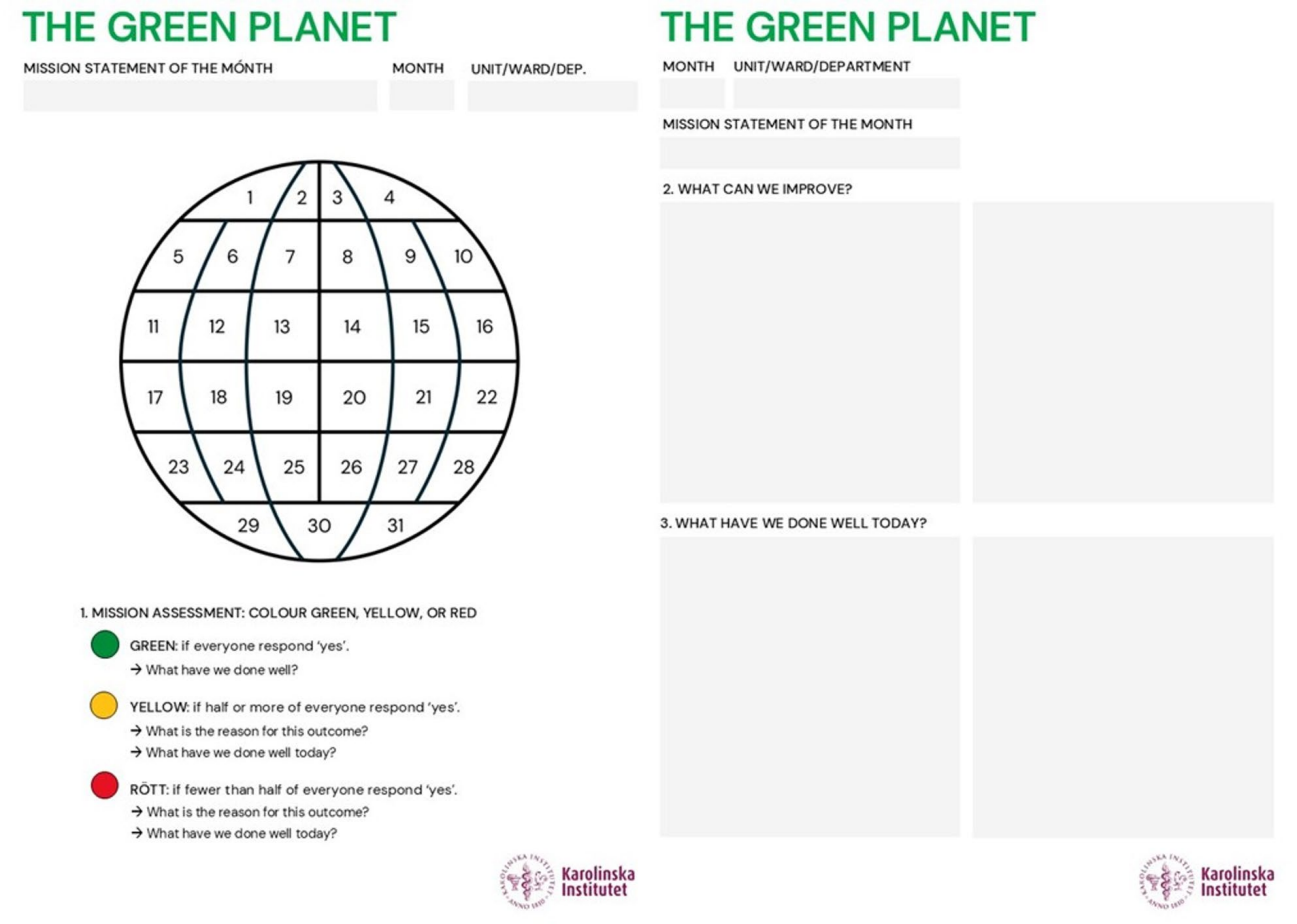
The printable template of the Green Planet.

The Green Planet is a visual method in which team members decide on a pro-climate mission (e.g., reducing the improper use of disposable gloves), thereby integrating climate actions into daily routine clinical work. During daily briefings near the Green Planet template (Fig. 2), the mission is assessed: if all respond “yes”, the corresponding date on the template is coloured green; if half or more respond “yes”, it is coloured yellow; and if fewer than half, or none, respond “yes”, it is coloured red. In addition, team members discuss climate “incidents”, and comments can be noted in the provided areas. At the end of the month, progress is summarised and discussed. Briefings and follow-up are encouraged to be integrated into regular meetings.

The Green Planet consists of: written instructions on one A4 page; a presentation, comprising a written script and PowerPoint slides; a printable template featuring a circle (a “planet”) divided into sections numbered 1–31 to represent the days of a month, and space for notes (Fig. 2); pro-climate missions developed based on workshops and the literature [5–7, 46, 47]; an information booklet outlining the research project, healthcare-related emissions, and their health consequences; and information about the test.

Putting the Green Planet into practice involves three phases: preparation conducted by a test leader in cooperation with a manager and/or colleagues; a one-month use of the Green Planet, during which staff work according to the set mission and participate in daily briefings led by a shift leader; and follow-up conducted by the test leader at the end of the test period. The activities for each phase are presented in the Results section (Table 1).

### Study of the intervention

The Green Planet was tested in an internal medicine ward with prior experience of using the Green Cross. A licenced practical nurse (LPN) working on the ward, who also served as an environmental representative and had been involved in the service design process (Fig. 1), suggested the ward for testing and volunteered as test leader. The ward had 24 beds and employed 23 registered nurses (RN) and 20 LPNs. The ward was led by one manager and one deputy manager, who both were RNs.

The evaluation of the intervention was guided by the COM-B model [28] that outlined how the Green Planet could promote PCBs by fostering capability, opportunity, and motivation [28].

### Data collection

Data was collected through semi-structured interviews, focus group discussion (FGD), and non-participatory observations between May and July 2025.

The focus was to explore participants’ experiences of using the Green Planet, and whether and how its use triggered the expected conditions based on the COM-B model [28]. Guides for the interviews and the FGD were developed for the study (Supplementary File 1) by authors LSS, DK, and PM who contributed expertise in qualitative methods, ethnology, sustainable development, healthcare management, and organisational psychology.

Eligible participants included the manager and staff exposed to the Green Planet who were working during the data collection period (i.e., day and evening shifts), reflecting a convenience sampling approach.

Eight individual interviews were conducted with 3 RNs, 3 LPNs, and the test leader who was interviewed twice: once during and once after the test. This sample thereby represented the range of professional roles within the ward. Six interviewees were female and one was male. The test leader was recruited for interviews prior to the test. RNs and LPNs were recruited by the researchers during the test, either in conjunction or following observations of briefing meetings or other ward meetings. The interviews were conducted in a staff room at the ward by DK and the service design expert and lasted between 5 and 30 min (two lasted less than 10 min, six lasted 11–19 min, and two lasted more than 20 min).

A FGD was conducted after the test and included eleven participants: RNs, LPNs, and the ward manager. Participants were recruited by the test leader, who invited scheduled staff to consider participating. The FGD was led by LSS, with assistance from DK, and lasted approximately 30 min. All interviews and the FGD were audio-recorded and transcribed verbatim.

Additionally, three non-participatory observation was conducted by DK, based on an observation scheme (Supplementary File 1), to gain understanding of how the briefings were conducted. Field notes were taken.

### Data analysis

For the first research question, the data was analysed using inductive content analysis [48]. The transcripts were read several times, and initial codes were generated (LSS and DK). Text extracts corresponding to these codes were identified and selected as illustrative excerpts. Microsoft Excel was used to organise the codes and associated text extracts (DK). The codes were then grouped into categories (LSS and DK) using an online platform (miro.com). These categories were subsequently mapped in relation to the intended use of the intervention (LSS, DK, and PM).

For the second research question, deductive content analysis [48], based on the COM-B model [28], was applied. A codebook derived from the model [28] was developed. The data was read multiple times, discussed, and coded through a collaborative process (LSS and DK). Microsoft Excel was used. Codes were then sorted into the a priori defined COM-B components and domains (LSS, DK, and PM), and this sorting was iteratively refined into inductively developed categories using the online platform (miro.com).

## Results

The results are organised in two sections: how the Green Planet was used and experienced (RQ 1) and if and how it influenced the conditions (capability, opportunity, motivation) for PCBs (RQ 2).

### Use of the Green Planet (RQ1)

Overall, the Green Planet method was implemented largely in line with the provided instructions and intended use, although some adaptations were made to better suit the local context. The main deviations between the intended use and its use in practice are presented in Table 1. The preparation phase was conducted largely as expected. During the use phase, deviations were observed in relation to the roles assumed by the shift leader and the test leader in facilitating the daily briefings, as well as in how reflections and mission assessments were conducted. Two missions were selected for the test: reducing the improper use of disposable gloves during the first month and reducing the improper use of single use aprons during the second month. The evaluation reported in this article focuses on the first test period.

**Table 1:** Planned and actual use of the Green Planet

Phases	Intendent use of the Green Planet	The use of the Green Planet in practice
Preparation	Decision on mission statement by test leader alone, in collaboration with management, and/or in collaboration with staff.	As expected.
	Presentation of the Green Planet mission at a staff meeting.	As expected.
	Display the printed template in a visible location.	As expected. Green Planet was displayed on a whiteboard alongside additional elements such as reflection questions, improvement templates, and visual decorations created by staff.
Use	Staff reflect on the mission and act in accordance with its goals throughout their shifts.	As expected, but with varying engagement from different staff members. Less engagement among some staff members was perceived be due to physical fatigue, lack of interest and/or time, high staff turnover or working part-time. The test leader provided occasional reminders during shifts, at daily briefings, at staff meeting, and even on social media, to reinforce the mission and encourage reflection.
	Daily briefings led by the shift leader and held near the Green Planet template.	Partly as expected. The responsibility for leading daily briefings was inconsistently fulfilled by the shift leader at the beginning of the test period. When the test leader was scheduled to work, the test leader often had to take on the role instead of the shift leader. Otherwise, the manager occasionally assisted in performing the briefings. If neither were available, briefings were generally not conducted in the beginning of the test period. However, several shift leaders started to perform briefings at later stages of the test period. The sessions were held in the staff common room rather than near the Green Planet template as instructed.
	Mission assessment during daily briefings held by the shift leader and reflection documented using colour coding and guiding questions (Fig. 2). Briefings include three steps: First, the success of the mission is assessed: if all staff respond “yes”, the corresponding date on the Planet is coloured green; if half or more respond “yes”, it is coloured yellow; and if fewer than half, or none, responded “yes”, it is coloured red. Second, staff reflect on what could be improved to better achieve the mission. Third, they reflect on what had gone well.	Partly as expected. During periods of high patient pressure, formal briefings were replaced by informal individual check-ins conducted by the test leader. At the end of the first test, the Green Planet template had been completed on 18 out of 31 days (16 green, 2 yellow), with unfilled days mainly attributed to the test leader’s absence and limited staff engagement. The test leader and manager occasionally introduced their own prompts and case-based discussions. Reflections were documented on two occasions: once directly on the template and once via a post-it note.
Follow-up	At the end of the month, the Green Planet progress is summarised and discussed during one established meeting by the test leader together with staff. The test leader leads the reflection regarding the results, experiences, areas for improvement, and future missions.	Partly as expected. No formal summative analysis or group reflection was conducted, but some informal feedback and observations were shared among staff. Second mission was selected. Two LPNs suggested reducing single-use aprons to be the mission for the second month, which the test leader adopted.
	The Green Planet template was to be saved physically or digitally.	As expected.

Overall, participants reported a positive experience of using the Green Planet. Several described a shift in their approach to glove use. Specifically, one participant noted that glove supplies were not depleted as quickly as before, suggesting reduced consumption, while another observed that colleagues had stopped using gloves in situations where they were not needed. However, other staff members pointed out that a one-month time period was insufficient to observe tangible results.

Staff expressed support for the continued use of the Green Planet. Plans were already in place to extend its use within the ward, and participants were in favour of adapting and implementing the method in other wards. Overall, the initiative was seen as promising, although several areas for improvement were also identified.

### Experiences of how the Green Planet influenced the conditions for PCBs (RQ2)

**Fig. 3:**
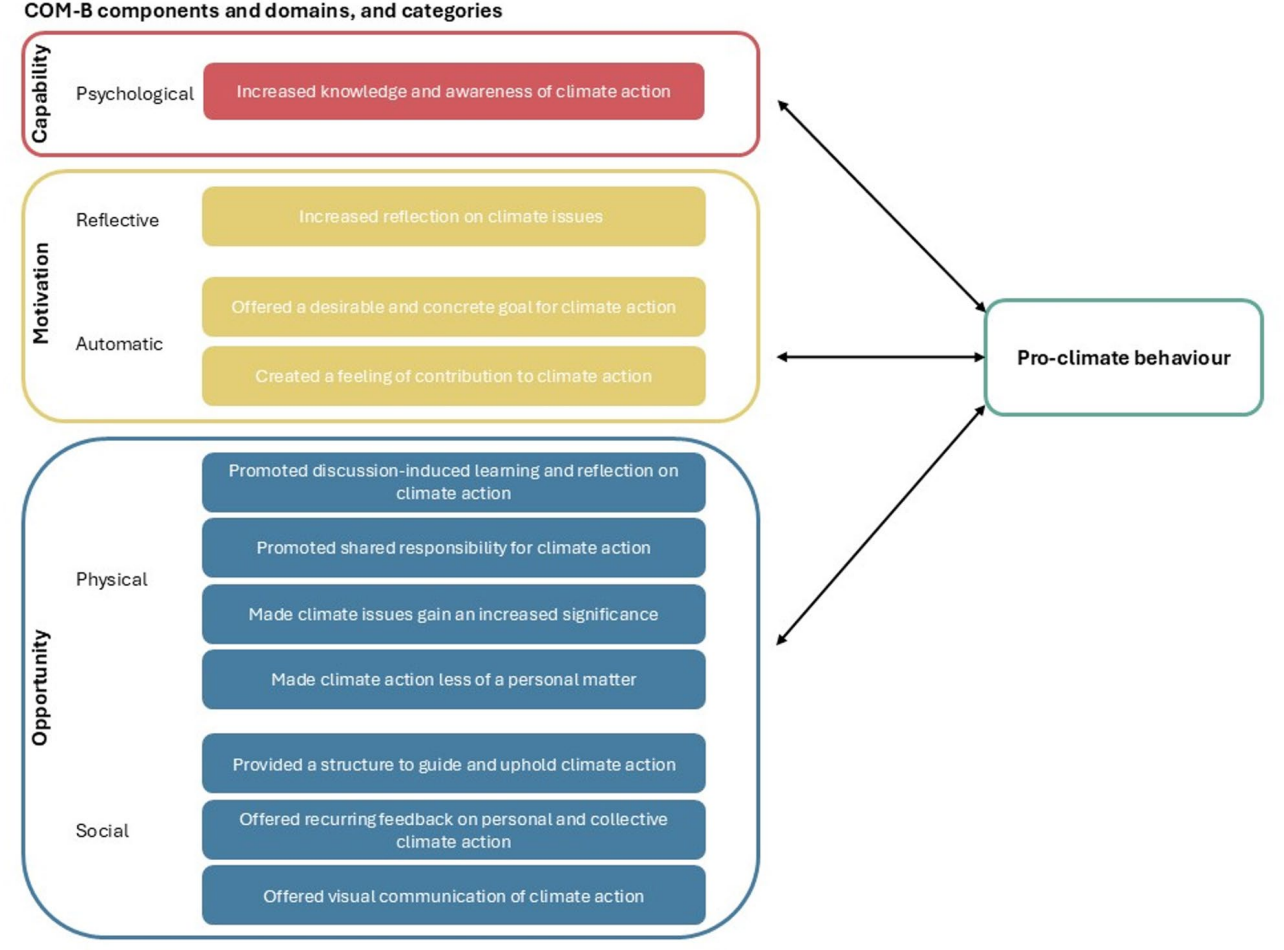
Experiences of how the Green Planet influenced conditions for pro-climate behaviours.

By analysing participants’ experiences through the COM-B model, the Green Planet appeared to improve the conditions for PCBs across the components (i.e., capability, motivation, and opportunity), and their respective domains: psychological capability, reflective and automatic motivation, and social and physical opportunity (Fig. 3). No influence was identified on the domain of physical capability. The results are further elaborated through eleven categories.

#### Capability: psychological

Participants experienced the Green Planet to increase knowledge and awareness of climate actions, and thereby, their psychological capability was enhanced.

##### Increased knowledge and awareness of climate action

Participants reported that the Green Planet increased their knowledge of appropriate glove use. Knowledge sharing occurred both during briefings and in daily practice, i.e., while performing clinical tasks.

*I have noticed a difference. Before*,* you might wear gloves just to*,* for example*,* check-up on the patient … Now that the Green Planet has been introduced*,* they [staff] have been taught and informed that this is not correct and not needed at all. So*,* knowledge about this has increased*,* and you can see a difference*. [RN01]

The Green Planet method also increased awareness of climate issues. Participants described how the method reminded them of the climate issues connected to the mission, making these issues more difficult to overlook.

*I think that before [we worked with climate issues]*,* these issues were not raised as actively. Talking about them every day makes the invisible visible. Talking about them actively brings it to life. Otherwise*,* they remain somewhere in the back of your mind*,* where they are not as vivid because you do not address them in the same way. … You may then overlook them or not take them as seriously*,* because you do not discuss it as deeply or bring them up every day*. [RN03]

Despite the perception that the Green Planet had contributed to increased knowledge of climate issues, participants suggested incorporating additional educational components. For example, they proposed brief introductions explaining the purpose of the mission and its connection to the hospital’s broader climate goals.

#### Motivation: reflective & automatic

The Green Planet method was experienced to help reflecting on the climate impact of work practices and to provide a desirable and concrete goal for climate action, thus enhancing reflective motivation. Participants also described a sense of being able to contribute to climate action, which can be interpreted as a form of automatic motivation.

##### Increased reflection on climate issues

Participants experienced that the Green Planet influenced their approach to climate actions within the ward. Although engagement with climate issue varied among staff, participants noted that they more frequently reflected on how their clinical practice, for example, the use of gloves and disposable items, contributed to excessive waste.

*I think it has had a quite positive impact because*,* in healthcare*,* you do not always think about how much you use gloves*,* aprons*,* and so on. You throw away more than necessary. So*,* I think it has had a pretty good impact*,* because now more people stop and think*,* rather than just grabbing a pair of gloves. They think*,* ‘Do I really need them right now?’ So*,* yes*,* I think it has been quite positive. People are thinking about it more.* [RN01]

Furthermore, participants described that the Green Planet encouraged them to reflect on their PCBs beyond the workplace.

*No*,* we probably have not thought about it [climate issues] that much before we started working with the Green Planet. So*,* then you must think for yourself*,* and I do not really reflect on environmental impact in my everyday life. But when it comes up*,* it affects you in a way that you start thinking about it*,* and then maybe you also begin to think about it at home*,* about why you do things.* [LPN02]

The Green Planet mission was believed to stimulate reflection on PCBs beyond the scope of the selected mission, for example in relation to recycling. The Green Planet was also experienced as making climate issues more salient and harder to overlook. Participants reasoned that even when the mission focused on reducing glove use, they reflected on how their behaviours could have broader environmental impact.

##### Offered a desirable and concrete goal for climate action

The Green Planet provided a desirable and concrete goal to strive for, rather than leaving to each and every one to “think about the climate” in an abstract manner. The climate mission created a shared goal that felt easier to manage, as staff could remind one another of the mission. Participants also described that it was in everyone’s interest to “become green”, which fostered motivation and willingness to participate.

*I have noticed a difference*,* in that we more often say to each other things like*,* ‘Remember that we have Green Planet’*,* because before we did not really have anything to relate it [climate action] to. Now we can remind each other in a different way than we might have done before. Previously*,* we might not have said anything*,* now it is more*,* ‘Remember that we are striving to become green’ - because everyone wants us to be green.* [RN02]

However, some participants suggested that both the mission and its impact on climate could be clarified further, for example by including numerical data to make assessments more objective.

##### Created a feeling of contribution to climate action

The Green Planet method offered a way for participants to feel actively involved in climate action and that they were doing something concrete to contribute to climate change mitigation. Participants explicitly stating that they found the process enjoyable.

*I think it is good [the experienced use of the Green Planet]. I think it is fun that we are doing something about it [climate change]*,* because otherwise it feels like it is just this ‘think about the environment’ that you always say*,* but then not much is done.* [RN02]

Participants explained that their behaviours could have an impact in the bigger picture, emphasising that actions within their clinical practise can make a difference.

*I think that employees*,* like all healthcare staff*,* feel that they are contributing something good. And that we are doing something … that we have something concrete with this environmental [method]. Now we actually use less gloves*,* but you also need to measure and see if that really is the case. But*,* just taking the first step*,* starting to reflect on it*,* and actively doing something will lead to less unnecessary use of gloves.* [RN03]

#### Opportunity: social & physical

The Green Planet fostered conversations and collaboration among staff regarding climate action, giving the issue a more prominent role within the ward, which in turn enhanced social opportunity for PCBs. It was also mentioned how the Green Planet offered a way for climate action to become part of routine work tasks rather than being a personal remark. The Green Planet also provided a structure to guide and sustain climate action and the template (Fig. 2) provided recurring visual feedback which created a physical opportunity for PCBs.

##### Promoted discussion-induced learning and reflection on climate action

The Green Planet promoted more conversations among staff, which facilitated learning and reflection on climate action. These discussions occurred both spontaneously between staff members and during the briefings led by the shift leader or the test leader.

Staff helped one another when uncertainties regarding correct glove use arose and could jointly reason to reach solutions. One participant had observed another staff member pointing out to a colleague that gloves were not necessary for a particular task. The two colleagues then reflected together and agreed that glove use was unnecessary, which subsequently resulted in a “yellow day” on the Green Planet template.

*I have seen it once during these six weeks. That someone that kind of did ‘no*,* wait*,* you do not need this’. And then they kind of reflected together*,* ‘oh*,* right*,* that is true’*,* and then we became yellow for like a day. Then we have kind of come to terms with us being yellow because we … Sometimes you automatically put on gloves when you do not need to.* [LPN, FGD]

Reflections could also arise during the briefings, regardless of the success of the mission, and staff occasionally engaged in case-based discussions about correct actions to fulfil the mission. The shift leader or test leader could pose open-ended questions to the group and facilitate discussions when there were differing opinions about the practice.

*We have our briefings*,* and we go through whether we feel we have used it in the right way. And when we should use it. And then we can also bring up different situations and then have a discussion with ‘how would you think here?’ if we say that… ‘If I am going to go in and take a patient’s blood pressure*,* would you use gloves then?’ So*,* we have more open questions. And then we can sort of discuss whether there is someone who says yes*,* someone who says no …* [RN02]

##### Promoted shared responsibility for climate action

The Green Planet promoted a sense of shared responsibility for climate actions. For instance, the test leader observed that several staff members became highly engaged in the Green Planet and initiated discussions with the test leader about potential future missions and other projects to reduce their environmental impact.

*I have colleagues who have come up with ideas*,* ‘this is something that we should be able to do to reduce climate impact’ or … there is a lot of thinking about paper. That we can laminate them. And then you can erase. And then you can use them several times.* [LPN, test leader, first interview]

Despite the observed increase in collaboration and engagement in climate actions among staff, the test leader occasionally felt isolated in their responsibility for managing the Green Planet. When on duty, the test leader often carried out briefings even when not assigned as shift leader, as other staff tended to perceive the Green Planet as solely the test leader’s responsibility.

##### Made climate issues gain an increased significance

Participants explained that, prior to the introduction of the Green Planet, discussions about workplace improvements were primarily focused on issues other than environmental aspects. For example, one participant described how climate action had previously been limited to occasional reminders from the environmental representative, which did not lead to further reflection on the issue. However, during the use of Green Planet, climate action became more integrated into everyday clinical work.

*Before … I do not know … there was not much [work with] climate [actions]. We did not think much about the climate [climate was not a priority]*,* it was more about economic issues*,* and then maybe … our environmental representative came and nagged us a bit about the environment*,* but I do not think we thought about it much before that actually.* [RN01]

##### Made climate action less of a personal matter

The Green Planet made it easier to both give and receive feedback about PCBs, as such feedback could be framed in relation to the fulfilment of the Green Planet mission rather than personal remarks.

*… the use of it [the Green Planet] is partly that if you can say to a colleague like this - ‘how is the glove use?’ And since they know that we’re working with the green planet*,* it won’t be so personal*,* but it will be easier to talk to each other and remind each other about the planet.* [LPN02]

*Then maybe now … when we have this Green Planet*,* I dare say to my colleagues a little more*,* ‘Yes*,* but think about this’*,* and then you have an excuse for why you say so*,* because sometimes people can take offence when you try to correct them a little … yes.* [RN01]

##### Provided a structure to guide and uphold climate action

The Green Planet mission was experienced to make climate actions more concrete compared to the past. Having a monthly focus, trackable progress, and daily briefings all contributed to reminding participants of the mission and stimulated discussion about climate action.

*I think we are more reminded of this when we have a focus area each month. Because if we do not have that*,* there is no discussion about it.* [RN02]

##### Offered recurring feedback on personal and collective climate action

The daily briefing process was argued, by some participants, to be more beneficial than weekly or monthly check-ins, as it provided immediate feedback that could be used to adjust actions. Although briefings were not always conducted as expected, the method was still argued to offer a way to identify shortcomings or challenges in performing the climate actions.

*… And then that you fill in every day and you check it like every twenty-four hours*,* not just like this once a week we check. But that you check every day. And so*,* it is the whole month*,* so it also becomes clear whether we are on the right track or not*,* I think that is super good about it.* [LPN02]

Reasons for daily briefings not being conducted as expected were the absence of the shift or team leader, staff reporting physical fatigue, or new staff being unaware of the Green Planet.

##### Offered visual communication of climate action

The Green Planet action sheets were placed on a whiteboard next to the Green Cross. Participants found that the use of distinct colours to communicate mission success was a simple way to track the day-to-day progress.

*What I think has worked well is that it is very clear what we should do. … It is very visible. We have a board*,* and we go through it every evening … And now we went through it just half an hour ago and it was green.* [RN03]

## Discussion

This study contributes an example of a concrete workplace intervention, the Green Planet, that was co-created with staff and managers in a Swedish hospital to foster PCBs. Participants had an overall positive experience of using the Green Planet and several conditions for PCBs were enhanced: by increasing knowledge and awareness of climate actions, promoting reflection on climate issues, and providing a desirable goal alongside a sense of contributing to climate action. Climate actions gained a larger role, as the Green Planet provided a structured approach supported by visual communication and recurring feedback. While this study only reports on initial experiences from a single ward, it offers several insights into the potential of the Green Planet as a method for supporting PCBs in healthcare settings.

### Creating conditions for PCBs through the Green Planet

The Green Planet builds on an established method for patient safety, the Green Cross [43]. The experiences of staff and manager suggest that the Green Planet can improve all conditions for PCBs, i.e., capability, motivation, and opportunity, as conceptualised in the COM-B model [28].

The Green Planet improved capability, by contributing to increased knowledge and awareness of the targeted climate mission, promoted through spontaneous conversations and structured daily briefings. These results mirror previous studies of the Green Cross method, which has been shown to increase knowledge and awareness of patient safety [43, 44]. Limited knowledge and awareness among healthcare workers is commonly described as a barrier to sustainable practises in healthcare [20, 23, 49]. Therefore, the Green Planet may represent a valuable approach to addressing this common barrier in healthcare climate mitigation.

The Green Planet also influenced automatic motivation. The method fostered a sense of contribution, partly facilitated by the presence of a desirable and concrete goal. Staff reported feeling more involved in climate action and that their actions clearly were connected to a larger purpose. Several psychological factors may otherwise hamper motivation to engage in climate action. For example, healthcare workers may feel disempowered to participate in mitigation efforts if they perceive their actions as having limited impact [50, 51]. Although the Green Planet did not provide objective data on the impact of the selected mission, which was identified as an area for improvement, it nevertheless provided participants with a desirable goal to strive for (the mission). The use of concrete targets on grass root level has been emphasized to promote climate action [52]. Thus, if combined with data on the effects of the mission selected, the Green Planet appears to be a promising method for fostering motivation. The mission itself, along with the visual feedback provided by the Green Planet, may also help reduce the perception of climate issues as abstract and distant (cf [25, 53]).

The method enhanced reflective motivation regarding climate action, both connected to clinical practice and beyond the workplace. Reflections on climate actions facilitated by using the Green Planet were hypothesised by the participants to have a spillover effect outside of the workplace. Positive spillover between PCBs is a common occurrence, especially when these behaviours require similar resources [54]. While previous studies have shown that personal engagement in climate action is seldom translated into workplace practices [10, 11], our results suggest that engagement in workplace climate actions may have a counter wise effect – from the professional role to a personal commitment.

Related to opportunity, the Green Planet created greater physical opportunities to guide and uphold climate action in clinical practises. While the method could not address the scarcity of time, which is an often-experienced barrier to pursue climate action [10, 20, 55], the Green Planet may help make better use of time by providing a structured approach within the clinical setting.

The Green Planet also enhanced social opportunity for PCBs by making climate actions a more collective effort. While the test leader and the shift leader held the overarching responsibility for the pilot test and the briefings, the responsibility for climate actions became more widely shared among staff. Limited staff commitment in environmental issues has been described as a barrier on all levels within healthcare organisations [56]. While appointing dedicated individuals (e.g. a change agent) or committees (e.g. green team) could address this issue [10, 24], these roles may still experience inadequate conditions [10]. Thus, future studies could explore if and how the Green Planet may be a viable way to make climate actions a collective responsibility while still having dedicated roles accountable to facilitate climate actions.

Opportunity, both physical and social, for PCBs were further strengthened through daily briefings involving staff, as these created a space for learning about climate actions. Visual and trackable presentation of progress in the Green Planet template, offered daily and instant feedback on the success of the mission. Similar results have been reported for the Green Cross method [43], which has been suggested to promote a learning environment through its collaborative elements such as interprofessional reflection, as well as the collection and discussion of data in near real time [45]. These findings are further corroborated by a systematic review showing improvement in quality indicators associated with feedback interventions [57]. The systematic review also found that feedback interventions were to be more successful when supplemented with post-feedback consultations, reminders, education, and decision support action toolboxes with pre-defined behaviours [57]. Similarly, the participants in our study suggested that the Green Planet could be complemented with, for instance, educational elements, suggesting that future studies could further explore what interventions could complement the Green Planet to foster PCBs, along with their long-term sustainability. Such studies would contribute to the limited and fragmented knowledge base on effective interventions for PCBs in healthcare [17, 18]. 

### The value for co-creating interventions for PCBs and integrating them into existing practices

The intervention examined in this study was co-created with staff and managers in a hospital setting using service design [35]. By actively involving end users throughout the development process, the Green Planet was grounded in everyday clinical realities, enhancing its relevance, acceptability, and feasibility in practice. The intervention also built on an established method, the Green Cross, which likely supported its uptake and integration into existing routines. This supports the assumption that staff and manager value the ability to build on existing routines and processes [30] rather than developing separate practices for climate action.

The integration of sustainability aspects into Quality Improvement (QI) processes has been previously suggested in the literature [58]. One example is the Sustainable Quality Improvement (SusQI) framework, which has been proposed as a way to assess the sustainability of QI initiatives by balancing patient and population health outcomes with their environmental, social, and financial impacts [58]. In this study, we have shown an additional area in which previously established QI methods, such as the Green Cross, can be adapted for climate action in clinical settings. More empirical studies are therefore needed to explore how QI methods, such as Green Planet, can support the integration of climate actions into healthcare, as well as how to measure observed changes in PCBs.

### Strengths and limitations

The study was informed by the COM-B framework, providing a theoretical ground for both intervention development and analysis, as well as contributing to the limited body of theory-informed research on PCB changes in healthcare [17, 18]. Another strength is the qualitative exploration of how the intervention was used in practice and how it influenced conditions for PCBs. This approach enabled insights into mechanisms of action, perceived benefits, and contextual factors that are often overlooked in quantitative evaluations of sustainability interventions. A further strength was the triangulation of three data sources.

Several limitations should be considered. As participation in the study was voluntary, the sample may have been influenced by self-selection bias, and no specific strategy was applied to ensure inclusion of staff with differing attitudes towards PCBs. Data was collected during daytime, missing night staff experiences. The FGD included many participants which might have influenced the depth of the data. Nevertheless, the varying data reflected varying perspectives on the intervention. Furthermore, the study was conducted in one ward which restricts transferability to other contexts with different organisational cultures, resources, or sustainability priorities. As the study focused on perceived changes in conditions for PCBs rather than measures of behaviour change or environmental impact, conclusions regarding actual reductions in GHG emissions cannot be drawn. Finally, as an early evaluation, the study did not assess long-term sustainability or maintenance of the intervention over time.

### Recommendations for future research

The findings of this study open several suggestions for future studies. Firstly, it would be valuable to evaluate the Green Planet intervention in more wards, a wider range of healthcare settings, including different clinical specialties, organisational contexts, and healthcare systems. Comparative studies across settings could help identify which contextual factors are most critical for successful implementation and sustained use.

Further, longitudinal studies are needed to examine whether the observed changes in capability, opportunity, and motivation translate into sustained PCBs over time. Future evaluations could incorporate quantitative measures, such as observed behaviour changes, or estimated GHG emissions, to complement qualitative insights and strengthen evidence of impact. Further research could explore how the Green Planet interacts with other complementary interventions.

### Implications for policy and practice

The findings suggest that PCB in healthcare can be supported through adaptable interventions that are embedded in everyday work and co-created with staff and managers. For practice, the Green Planet illustrates how climate action can be made visible, concrete, and collective, rather than abstract or individually burdensome. Healthcare organisations seeking to advance sustainability may benefit from adopting similar approaches that provide structure, feedback, and shared goals, while allowing flexibility for local adaptation.

For policy, the study highlights the importance of complementing formal sustainability and climate goals and policies with practical tools that enable staff engagement at the frontline. Policymakers and healthcare leaders should recognise that pro-climate behaviours are influenced not only by knowledge and motivation, but also by social norms, opportunities, and organisational support. Supporting participatory, theory-informed interventions may help bridge the persistent gap between climate ambitions and everyday clinical practice.

After completion of this study, the Green Planet was presented to four additional hospitals and received a positive response, as evidenced by the fact that several wards, clinics, and departments wished to test and implement the method. The method has also been included in the strategic plans of the hospital in which it was developed as well as in other hospital departments.

## Conclusions

This study demonstrates how a co-created, theory-informed intervention can be integrated into clinical work to support conditions for PCB among healthcare professionals. The Green Planet provided a shared structure that increased awareness, reflection, collaboration, and a sense of collective contribution to climate action. While findings do not establish direct environmental impact, these offer valuable insights into how workplace interventions can enable and normalise climate action in healthcare settings.

By focusing on everyday practices and involving staff and managers in the design process, the Green Planet shows promise as a scalable and adaptable approach to embedding sustainability into routine care. These findings contribute to the growing, but still limited, evidence base on behavioural interventions for climate action in healthcare. Further, it underscores the need for continued research that links intervention design, implementation, and long-term environmental outcomes.

## Supplementary Information

Below is the link to the electronic supplementary material.

Supplementary Material 1

## Data Availability

The dataset generated and analysed during the current study are not publicly available due to participant anonymity, ethical reasons, and data protection but are available from the corresponding author on reasonable request.

## Abbreviations

COM-B: Capability, Opportunity, Motivation–Behaviour model
FGD: Focus group discussion
LPN: Licenced practical nurses
PCB: Pro-climate behaviours
QI: Quality Improvement
RN: Registered nurses
SQUIRE: Standards for Quality Improvement Reporting Excellence
SusQI: The Sustainable Quality Improvement Framework
