# Supplementary material for: The Green Planet: development and early evaluation of a novel method for climate actions in clinical practice
Source: BMC Health Serv Res. 2026 Jul 2;26:909. doi: 10.1186/s12913-026-15059-z (PMC13330229; doi:10.1186/s12913-026-15059-z)
Supplement: Supplementary file 1 — Supplementary Material 1 [file 12913_2026_15059_MOESM1_ESM.docx]

1. Interview guide
2. Focus group discussion guide
3. Observation scheme
4. **Interview guide**

Can you describe how you work with the Green Planet at your workplace?

How has the Green Planet influenced how you work with climate-related issues at your workplace? In what way? Can you give examples?

- How does this differ from how you worked with climate issues before the Green Planet?

How has the Green Planet affected your daily work? In what way? Can you give examples? What was it like before (differences/similarities)?

How do you experience the use of the Green Planet?

How could the Green Planet be improved?

What works well with the Green Planet?

1. **Focus group discussion guide**

| Working with the Green Planet | Could you briefly describe how you have worked with the Green Planet? |
| --- | --- |
| Experiences of the Green Planet | How has it been for you to work with the Green Planet?  What obstacles have you encountered in making the entire planet turn green?  Has there been anything about the Green Planet that has been less good or even problematic? This can be based on your own experience or something you have heard from others.  What about the Green Planet has felt positive or worked well?  Is there anything about the Green Planet that makes it motivating to work with? *Explore what feels motivating (for example: direct feedback, its visibility, striving toward a goal, working together, links to patient safety/hygiene, etc.).* |
| Outcomes of the Green Planet | Can you describe whether the Green Planet has influenced how you work with climate issues at the workplace, and if so, how? Can you give examples? Has anything changed compared to how you worked with climate issues before?  How has the Green Planet affected your conditions for reducing the hospital’s climate impact?  How has the Green Planet influenced your ability to take action to reduce the hospital’s climate impact? For example, physical conditions, increased knowledge, or other factors?  How has the Green Planet affected your motivation to reduce the hospital’s climate impact?  How has the work with the [climate mission] influenced your actions? In what way?  Can you give examples of concrete behaviour changes in yourself or others? What was it like before (differences/similarities)?  Do you think that working with the [climate mission] has influenced your daily work in any way? In what way? Can you give examples? What was it like before (differences/similarities)?  Has using the Green Planet led you to think more about other climate‑related issues beyond the specific climate mission? |
| Finally | If you were responsible for the Green Planet and could make one change to improve it, what would you do?  Do you have any additional thoughts about the Green Planet that you would like to share? |

1. **Observation scheme**

|  |  |
| --- | --- |
| Date Time Place |  |
| Actor convening the meeting |  |
| Participating roles, invitees, and number of attendees |  |
| The reconciliation Start time Who starts the meeting Who leads the meeting  Content of the discussion What is said (positive and negative) Questions and concerns that arise  How many people speak, out of the total present  Description of what happens |  |
| Observer’s impressions |  |
